# PTEN Negatively Regulates MAPK Signaling during *Caenorhabditis elegans* Vulval Development

**DOI:** 10.1371/journal.pgen.1002881

**Published:** 2012-08-16

**Authors:** Itay Nakdimon, Michael Walser, Erika Fröhli, Alex Hajnal

**Affiliations:** 1 Institute of Molecular Life Sciences, University of Zürich, Zürich, Switzerland; 2 Cancer Network Zürich PhD Program, Institute of Molecular Life Sciences, University of Zürich, Zürich, Switzerland; 3 Molecular Life Sciences PhD Program, Institute of Molecular Life Sciences, University of Zürich, Zürich, Switzerland; Stanford University Medical Center, United States of America

## Abstract

Vulval development in *Caenorhabditis elegans* serves as an excellent model to examine the crosstalk between different conserved signaling pathways that are deregulated in human cancer. The concerted action of the RAS/MAPK, NOTCH, and WNT pathways determines an invariant pattern of cell fates in three vulval precursor cells. We have discovered a novel form of crosstalk between components of the Insulin and the RAS/MAPK pathways. The insulin receptor DAF-2 stimulates, while DAF-18 PTEN inhibits, RAS/MAPK signaling in the vulval precursor cells. Surprisingly, the inhibitory activity of DAF-18 PTEN on the RAS/MAPK pathway is partially independent of its PIP_3_ lipid phosphatase activity and does not involve further downstream components of the insulin pathway, such as AKT and DAF-16 FOXO. Genetic and biochemical analyses indicate that DAF-18 negatively regulates vulval induction by inhibiting MAPK activation. Thus, mutations in the PTEN tumor suppressor gene may result in the simultaneous hyper-activation of two oncogenic signaling pathways.

## Author Summary

The human tumor suppressor PTEN is mutated in many different types of cancer. Using the roundworm *C. elegans* as a model to study how cells communicate during animal development, we discovered a new mechanism by which PTEN inhibits the activity of the oncogenic RAS/MAPK signaling pathway. Focusing on the development of the vulva, the egg-laying organ of the hermaphrodite, as a model to investigate the regulation of RAS/MAPK signaling, we could distinguish between two distinct inhibitory activities of PTEN on the RAS/MAPK signaling pathway. On the one hand, PTEN acts as a lipid phosphatase that inhibits the production of PIP_3_, which in turn stimulates RAS/MAPK signaling. On the other hand, PTEN acts as a protein phosphatase that negatively regulates RAS/MAPK signaling by inhibiting signal transduction at the level of the MAPK, which is a key component in the pathway. Understanding the detailed molecular mechanism by which the PTEN tumor suppressor homolog regulates signal transduction in *C. elegans* can help predict the consequences of mutations in human PTEN for cancer development in humans.

## Introduction

PTEN (Phosphatase and TENsin homologue) is the second-most frequently somatically mutated tumor suppressor gene in human cancer. PTEN is often inactivated in glioblastoma, melanoma, prostate and endometrial neoplasia [1]. Germline mutations in PTEN are also known to cause a variety of rare syndromes, collectively known as the PTEN hamartoma tumor syndromes (PHTS) [2]. Cowden syndrome is the best-described syndrome within PHTS, with approximately 80% of patients carrying germline PTEN mutations [3]. The main reported function of PTEN is as a lipid phosphatase, which dephosphorylates PhosphatidylInositol(3,4,5)-trisphosphate (PIP_3_) at position 3, thereby inhibiting the insulin pathway [4]. However, PTEN can also act as a dual-specificity tyrosine and serine/threonine protein phosphatase [5], [6]. The catalytic phosphatase domain of PTEN (amino acids 123–131) contains several conserved amino acids, mutations of which affect the efficiency and specificity of the phosphatase activity [7]. One such mutation is G129E, which causes PTEN to lose its lipid phosphatase activity while retaining most of its protein phosphatase activity [4], [5]. Using the G129E mutation, numerous reports have provided evidence for a crucial role of PTEN protein phosphatase activity in regulating cell migration, invasion and spreading independently of the canonical insulin signaling pathway. For example, PTEN G129E regulates cell migration, spreading, and the formation of focal adhesions [8]. Moreover, PTEN G129E binds and de-phosphorylates the Focal Adhesion Kinase FAK *in vitro* [8]. In glioblastoma cells injected into nude mice, PTEN G129E expression inhibits cell invasion, accompanied by decreased FAK phosphorylation without changing the activity of the AKT kinase [6], [9]. Additionally, PTEN binds and dephosphorylates the adaptor protein Shc to modulate cell motility [10].

The *daf-18* gene encodes the single PTEN ortholog in *C. elegans* [11]. Under favorable growth conditions, *C. elegans* larvae pass through four distinct larval stages termed L1 to L4. However, under conditions of starvation or overcrowding, the L1 larvae enter a long-lived, stress resistant alternative developmental stage called the dauer larva stage. DAF-18 PTEN controls entry into the larval dauer stage, life span, neurite outgrowth and cell-cycle progression, mainly by inhibiting the insulin signaling pathway [12]–[14]. Human PTEN can functionally replace *C. elegans* DAF-18 to rescue the *daf-18(lf)* DAuer Formation defective (DAF-d) phenotype [15].

In the presence of abundant food, binding of various insulin ligands to the DAF-2 insulin receptor causes the activation of AGE-1, the only type I phosphatyidlinositol-3-kinase (PI3K) encoded by the *C. elegans* genome [11]. AGE-1 phosphorylates PI(4,5)P_2_ to PI(3,4,5)P_3_, which acts as a second messenger. PIP_3_ then activates the AKT-1 and AKT-2 kinases that phosphorylate and thereby inhibit the FOXO transcription factor DAF-16 [16]. In the absence of the insulin signal, non-phosphorylated DAF-16 enters the nucleus and activates genes promoting entry into the dauer stage [17]. The main reported function of DAF-18 PTEN is to antagonize the insulin pathway by dephosphorylating PIP_3_ [14]. Loss of *daf-18* thus leads to hyper-activation of the insulin pathway and a DAF-d phenotype, while loss of *daf-2* or *age-1* function leads to a DAuer Formation constitutive (DAF-c) phenotype.

Recent evidence indicates that similar to mammalian PTEN, *C. elegans* DAF-18 can also act as a protein phosphatase to regulate insulin-independent events. For example, DAF-18 PTEN directly binds and dephosphorylates the ephrin receptor tyrosine kinase VAB-1 to regulate oocyte maturation in the hermaphrodite germline [18]. Moreover, multiple genes causing synthetic lethality in combination with *daf-18(lf)* have been identified, pointing to additional functions of DAF-18 besides its role in insulin signaling [19].

The development of the *C. elegans* hermaphrodite vulva, the egg-laying organ, is one of the best-characterized models for organogenesis [20]. The interplay between the conserved RAS/MAPK, NOTCH, and WNT signaling pathways specifies two distinct vulval cell fates. Beginning in the L2 stage, the gonadal Anchor Cell (AC) releases the EGF ligand LIN-3, which activates the EGF receptor homolog LET-23 in the six Vulval Precursor Cells (VPCs). The VPC located nearest the AC (P6.p) receives most of the inductive LIN-3 EGF signal and hence exhibits the strongest activity of the RAS/MAPK pathway, allowing P6.p to adopt the primary (1°) vulval cell fate. Consequently, P6.p produces a lateral signal that activates the LIN-12 NOTCH signal in the neighboring VPCs P5.p and P7.p. Notch signaling in these two VPCs induces them to adopt the secondary (2°) cell fate and at the same time blocks transduction of the inductive LIN-3 signal by inhibiting MAPK activation. The 1° VPC P6.p and the 2° VPCs P5.p and P7.p each go through three rounds of cell division to generate 22 cells that form the vulva. The remaining three distal VPCs (P3.p, P4.p and P8.p) adopt the non-vulval 3° fate, which divide once and then fuse with the surrounding hypodermis (hyp7).

Mutations in genes encoding components of the RAS/MAPK, NOTCH, and WNT signaling pathways change the patterning of the VPC fates, which can readily be quantified. For example, mutations that hyperactivate the RAS/MAPK pathway cause the induction of more than three VPCs, resulting in a Multivulva (Muv) phenotype. On the other hand, mutations that cause a decrease in RAS/MAPK pathway activity, lead to the induction of fewer than three VPCs, a phenotype called Vulvaless (Vul).

In this work, we have discovered and characterized a new mode of crosstalk between components of the insulin and the RAS/MAPK pathways. Using genetic and biochemical epistasis analyses, we found that the insulin receptor DAF-2 stimulates while DAF-18 PTEN inhibits RAS/MAPK signaling in the VPCs. Surprisingly, part of the inhibitory activity of DAF-18 on the RAS/MAPK pathway is independent of its PIP_3_ lipid phosphatase activity and does not involve further downstream components of the insulin pathway. Our results indicate that DAF-18 negatively regulates vulval induction most likely by inhibiting MAP kinase MPK-1 signaling.

## Results

### *daf-18* inhibits vulval induction independently of the canonical insulin signaling pathway

In our previous work, we reported first evidence for a crosstalk between the DAF-2 insulin receptor and the RAS/MAPK signaling pathway during vulval development [21]. To further investigate this interaction, we performed a systematic epistasis analysis by combining various mutations in the insulin and RAS/MAPK signaling pathways and quantifying the levels of vulval induction (Table 1). As reported previously, a reduction-of-function *(rf)* mutation in the insulin receptor *daf-2* partially suppressed the Muv phenotype of *let-60 ras* gain-of-function *(gf)* animals (Table 1, rows 1, 2) [21]. Conversely, a *loss-of-function (lf)* mutation in the *PTEN* homolog *daf-18* strongly enhanced the *let-60(gf)* Muv phenotype (Table 1, row 3). Moreover, *daf-18(lf)* suppressed the vulvaless (Vul) phenotype caused by mutations in the EGF receptor *let-23* or in its cofactor *lin-2*, which activates the RAS/MAPK signaling pathway in the VPCs (Table 1, rows 4–7). Since DAF-18 PTEN counteracts the type I phosphatidyl-inositol-3 kinase (PI3K) AGE-1 that transduces the insulin signal downstream of DAF-2, we tested if an *age-1(lf)* mutation could revert the enhanced vulval induction caused by *daf-18(lf)*. Surprisingly, *age-1(lf)* only partially suppressed the increase in vulval induction caused by *daf-18(lf)*, both in the *let-60(gf)* and the *lin-2(lf)* backgrounds (Table 1, rows 8, 9). Also, the *daf-2(rf)* mutation only partially reverted the enhancement of the *let-60(gf)* Muv phenotype by *daf-18(lf)* (Table 1, row 10), suggesting that DAF-18 inhibits vulval induction to some extent independently of DAF-2 and AGE-1. However, mutations in further downstream components of the DAF-2 insulin pathway had no detectable effect on vulval induction. For example, a *gf* mutation in *akt-1*, which encodes one of the two AKT homologues transducing the insulin signal downstream of AGE-1 [16], did not suppress the *let-23(rf)* Vul phenotype (Table 1, row 11), and a *lf* mutation in *akt-1* did not suppress the *daf-18(lf) let-60(gf)* Muv phenotype (Table 1, row 12). To examine a possible redundancy between the two *akt* genes, we performed *akt-2* RNAi in *daf-18(lf) let-60(gf); akt-1(lf)* triple mutants, but observed no reduction in vulval induction compared to the RNAi controls (Table 2, rows 1, 2). Also, a *lf* mutation in *daf-16*, which encodes a FOXO transcription factor that is negatively regulated by the insulin pathway, did not enhance the *let-60(gf)* Muv phenotype (Table 1, row 13).

**Table 1:** Epistasis analysis between the insulin and RAS/MAPK pathways.

	genotype	Induction±SE	% Vul	% Muv	n
1	*let-60(gf)*	4.16±0.05	0.00	79.1	283
2	*daf-2(rf); let-60(gf)*	3.24±0.11*** ^(1)^	0.00	19.3	31
3	*daf-18(lf) let-60(gf)*	4.99±0.05*** ^(1)^	0.00	96.4	221
4	*let-23(rf)*	0.55±0.08	93.8	0.00	129
5	*let-23(rf); daf-18(lf)*	1.90±0.10*** ^(4)^	63.9	6.8	133
6	*lin-2(lf)*	1.34±0.19	73.8	0.00	42
7	*daf-18(lf); lin-2(lf)*	2.50±0.12*** ^(6)^	35.5	1.7	59
8	*age-1(lf); daf-18(lf) let-60(gf)*	4.61±0.08*** ^(1)^	0.00	94.7	113
9	*age-1(lf); daf-18(lf); lin-2(lf)*	2.15±0.16*** ^(6)^	53.1	6.1	49
10	*daf-2(rf); daf-18(lf) let-60(gf)*	4.40±0.17^(1)^	0.00	88.0	25
11	*let-23(rf); akt-1(gf)*	0.36±0.13^(4)^	94.9	0.00	39
12	*daf-18(lf) let-60(gf); akt-1(lf)*	4.80±0.29^(3)^	0.00	80	15
13	*daf-16(lf); let-60(gf)*	4.18±0.11^(1)^	0.00	77.5	80
14	*age-1(lf); let-60(gf)* †	4.08±0.06^(1)^	0.00	76.2	164
15	*age-1(lf); daf-16(lf); let-60(gf)*	3.62±0.15** ^(1)^	0.00	54.1	24

SE indicates the standard error of the mean. % Vul indicates the fraction of animals with less than three induced VPCs. % Muv indicates the fraction of animals with more than three induced VPCs. Induction indicates the average number of induced VPCs per animal.

** indicates a p-value≤0.005;

*** indicates a p-value≤0.0005. Numbers in brackets indicate the row against which a t-test was performed. Alleles used: LG I: *daf-16(mu86)*, LG II: *age-1(mg44), let-23(sy1)*, LG III: *daf-2(e1370)*, LG IV: *let-60(n1046), daf-18(ok480)*, LG V: *akt-1(ok525lf), akt-1(mg144af)*, LG X: *lin-2(n397)*.

† F1 progeny of heterozygous *age-1(lf)/+* parents.

**Table 2:** RNAi against *akt-2* and alternative PI3Ks.

	genotype	RNAi	Induction ±SE	% Vul	% Muv	n
1	*daf-18(lf) let-60(gf); akt-1(lf)*	*gfp*	5.41±0.12	0.00	100	17
2	*daf-18(lf) let-60(gf); akt-1(lf)*	*akt-2*	5.53±0.18	0.00	94.4	18
3	*age-1(lf);daf-18(lf);lin-2(lf)*	*gfp*	1.59±0.34	71	12	17
4	*age-1(lf);daf-18(lf);lin-2(lf)*	*vps-34*	1.60±0.28	65	0	20
5	*age-1(lf);daf-18(lf);lin-2(lf)*	*piki-1*	1.69±0.31	69	6	16
6	*age-1(lf);daf-18(lf);let-60(gf)*	*gfp*	5.14±0.20	0	94	17
7	*age-1(lf);daf-18(lf);let-60(gf)*	*vps-34*	5.40±0.16	0	100	15
8	*age-1(lf);daf-18(lf);let-60(gf)*	*piki-1*	5.44±0.12	0	100	17

SE indicates the standard error of the mean. % Vul indicates the fraction of animals with less than three induced VPCs. % Muv indicates the fraction of animals with more than three induced VPCs. Induction indicates the average number of induced VPCs per animal. Alleles used: LG II: *age-1(mg44)*, LG IV: *daf-18(ok480)*, *let-60(n1046)*, LGV: *akt-1(ok525lf)*, LG X: *lin-2(n397)*.

We further tested the role of AGE-1 during vulval development. Since *age-1(lf)* leads to a constitutive dauer phenotype (DAF-c) that is maternally rescued, homozygous *age-1(lf)* worms could only be analyzed in the F1 progeny of heterozygous *age-1(lf)/+* parents or when rescued by the *daf-16(lf)* mutation. Our analysis indicated that *age-1* also exhibits a partial maternal rescue in vulval induction since the homozygous *age-1(lf); let-60(gf)* progeny obtained from heterozygous *age-1(lf)/+* parents displayed similar levels of vulval induction as *let-60(gf)* single mutants (Table 1, row 14). In contrast, homozygous *age-1(lf); let-60(gf)* double mutants maintained in the *daf-16(lf)* background exhibited a partially suppressed Muv phenotype, though *age-1(lf)* suppressed the *let-60(gf)* Muv phenotype to a lesser extent than *daf-2(rf)* (Table 1, row 15, p-value≤0.05 compared to row 2).

Taken together, the genetic analysis indicates that the DAF-2 insulin receptor promotes and DAF-18 PTEN inhibits vulval induction. DAF-2 and DAF-18 both regulate vulval induction through AGE-1-dependent as well as AGE-1-independent pathways that do not utilize the canonical insulin pathway downstream of AGE-1.

### DAF-18 inhibits vulval induction independently of other PI3Ks

AGE-1 is the only *C. elegans* member of the type I family of PI3Ks, which convert PI(4,5)P_2_ into PI(3,4,5)P_3_. To further investigate the AGE-1-independent effect of DAF-18 on vulval induction, we tested the roles of alternative PI3Ks that can phosphorylate PIs at position 3. *vps-34* encodes a type III PI3K, which catalyzes the production of PI(3)P_1_, and *piki-1* encodes a type II PI3K involved in the engulfment of apoptotic cell corpses [22]. In order to examine the role of these alternative PI3Ks during vulval induction, we performed RNAi against *vps-34* and *piki-1* in *age-1(lf); daf-18(lf); lin-2(lf)* animals and tested for a further reduction of vulval induction. RNAi to *vps-34* and *piki-1* has been previously shown to be effective in different tissues [23], [24]. Neither *vps-34* nor *piki-1* RNAi caused any significant reduction in the number of induced VPCs when compared to control (*gfp*) RNAi animals (Table 2, rows 3–5). Furthermore, *vps-34* and *piki-1* RNAi in an *age-1(lf); daf-18(lf) let-60(gf)* background did not cause a decrease in vulval induction (Table 2, rows 6–8). It thus seems unlikely that an alternative PI3K acts redundantly with AGE-1 during vulval induction, further supporting our observation that DAF-18 regulates vulval induction not only by regulating PIP_3_ levels but also via a lipid phosphatase-independent activity.

### *daf-18* inhibits 1° vulval cell fate specification

To characterize the nature of the cell fate transformation caused by *daf-18(lf)*, we quantified the levels of the EGL-17::CFP reporter, whose expression is induced by RAS/MAPK signaling in the 1° vulval cell lineage [25] and of the LIP-1::GFP reporter whose expression is induced by LIN-12 NOTCH signaling in the 2° vulval cell lineage [26].

**Figure 1:**
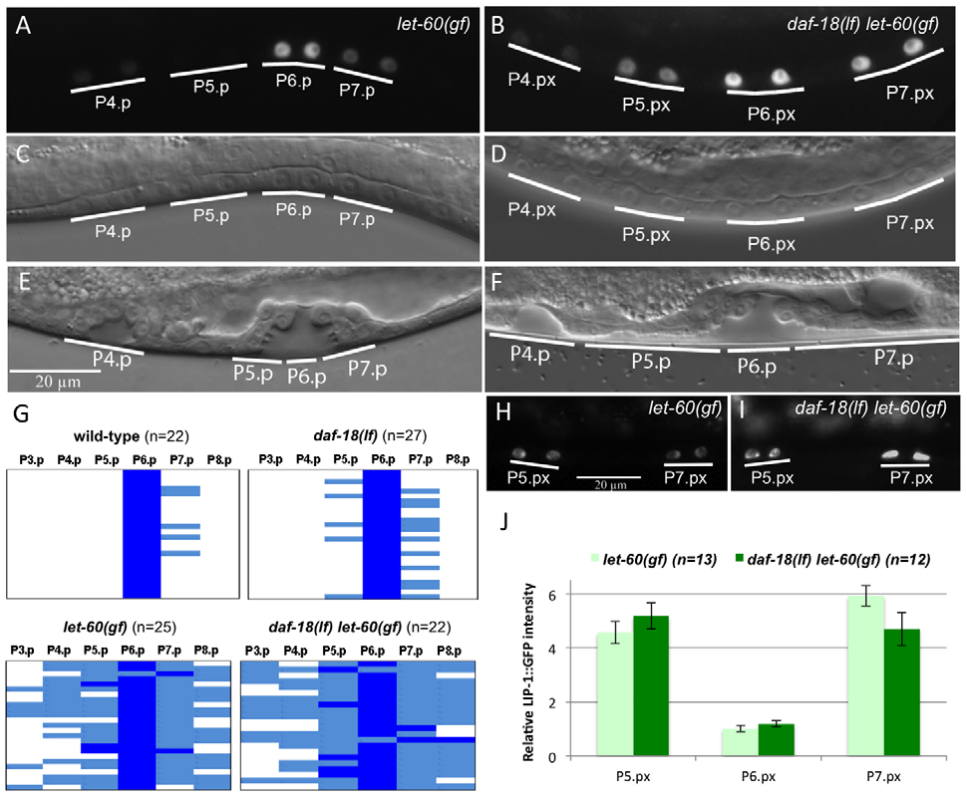
DAF-18 inhibits 1° vulval fate specification. (A) Expression of the 1° cell fate marker EGL-17::CFP in *let-60(gf)* single and (B) *daf-18(lf) let-60(gf)* double mutants at the Pn.px stage. (C and D) show the corresponding Nomarski images of the animals shown in (A) and (B), respectively. (E) Morphology of the vulval invagination in a *let-60(gf)* mutant at the Pn.pxxx stage. Note that the descendants of P5.p and P7.p remain attached to the cuticle, which is characteristic of the 2° cell fate. (F) P5.p and P7.p often detach from the cuticle in *daf-18(lf) let-60(gf)* animals, resulting in a broad invagination with abnormal morphology. (G) Quantification of the 1° fate marker EGL-17::CFP expression at the Pn.px and Pn.pxx stages in the indicated genetic backgrounds. Dark blue represents cells with high EGL-17::CFP expression, corresponding to at least 50% of the intensity observed in the P6.px(x) cells, light blue represents cells with clearly visible but less than 50% of the P6.px(x) signal, and white spaces represent cells with undetectable levels of EGL-17::CFP. (H) Expression of the 2° fate marker LIP-1::GFP in *let-60(gf)* and (I) *daf-18(lf) let-60(gf)* animals. (J) Quantification of LIP-1::GFP expression in the P5.p through P7.p descendants. Signal intensities are indicated relative to P6.px(x) in *let-60(gf)*.

*daf-18(lf) let-60(gf)* double mutants showed an increased frequency of adjacent VPC descendants expressing high levels of EGL-17::CFP when compared to *let-60(gf)* single mutants (Figure 1A–1D). Specifically, in *daf-18(lf); let-60(gf)* double mutants 27% of adjacent VPC descendants showed strong EGL-17::CFP expression (i.e. at least 50% of the signal intensity seen in the P6.p lineage) versus 16% in *let-60(gf)* single mutants (Figure 1G). Furthermore, while the 2° P5.p and P7.p descendants in the wild-type displayed weak (i.e. less than 50% of the P6.px signal intensity) EGL-17::CFP expression in 22% of the cases, 52% of *daf-18(lf)* single mutants showed EGL-17::CFP expression in the 2° cells (Figure 1G).

Besides the slight increase in EGL-17::CFP expression, the morphology of the vulval invagination at the L4 larval stage was changed in *daf-18(lf) let-60(gf)* double mutants. The vulval invagination formed by the P5.p to P7.p descendants of most *let-60(gf)* single mutants resembles the single invagination formed in the wild-type (Figure 1E). In *daf-18(lf) let-60(gf)* double mutants, on the other hand, the P5.p to P7.p descendants were often completely detached from the cuticle, resulting in an abnormal shape of the vulval invagination (Figure 1F) (37% detached P5.p and/or P7.p descendants in *daf-18(lf) let-60(gf)* versus 3% detached in *let-60(gf)*, n = 54 and n = 35, respectively). A detachment of the P5.p and P7.p descendants from the cuticle is indicative of a 2° to 1° cell fate transformation as it has been observed in mutants exhibiting elevated MAPK activity in the 2° lineage [27].

In contrast to the 1° fate marker EGL-17::CFP, expression of the 2° fate marker LIP-1::GFP was not changed in *daf-18(lf)* mutants. In particular, LIP-1::GFP levels in the P5.p and P7.p descendants were unchanged in *daf-18(lf) let-60(gf)* double mutants compared to *let-60(gf)* single mutants (Figure 1H–1J).

Thus, *daf-18(lf)* enhances specification of the 1° cell fate and causes a partial 2° to 1° fate transformation in P5.p and P7.p without affecting the strength of the lateral LIN-12 NOTCH signal.

### *daf-18* negatively regulates vulval induction downstream of *sos-1* and upstream or at the level of *mpk-1*

Since EGFR/RAS/MAPK signaling induces the 1° vulval cell fate and *daf-18(lf)* mutants exhibited an increased expression of the 1° cell fate marker EGL-17::CFP, we sought to determine at what level DAF-18 inhibits the activity of the EGFR/RAS/MAPK signaling pathway. For this purpose, we performed further epistasis analysis combining *daf-18(lf)* with mutations in different components of the RAS/MAPK pathway. Even though *daf-18(lf)* single mutants showed no obvious changes in the vulval fate pattern (Table 3, rows 1, 2), *daf-18(lf)* increased the levels of vulval induction in most of mutants in the RAS/MAPK pathway tested, confirming that DAF-18 negatively regulates the RAS/MAPK signaling during vulval induction. For example, when combined with mutations in different positive regulators of the RAS/MAPK pathway such as *let-23(rf)*, *lin-2(lf)* or *lin-45(rf), daf-18(lf)* significantly suppressed the Vul phenotype of these mutants (Table 3, rows 5–8 and 11–12). In particular, *daf-18(lf)* suppressed a *lf* mutation in the RAS-GEF *sos-1* when assayed in the *let-60(gf)* background to rescue the lethality caused by *sos-1(lf)*, placing *daf-18* function downstream of *sos-1* (table 3, rows 9–10). However, since vulval induction in *sos-1(lf); let-60(gf)* animals is partly sensitive to the inductive anchor cell signal [28], we cannot exclude the possibility that DAF-18 might inhibit RAS/MAPK signaling through a SOS-1 independent branch of the pathway. As an exception, *daf-18(lf)* did not suppress the Vul phenotype of *lin-3(rf)* mutants (Table 3, rows 3–4), suggesting that *daf-18(lf)* alone is not sufficient to activate the RAS/MAPK pathway in the absence of the inductive AC signal. Furthermore, *daf-18(lf)* did not affect the completely penetrant Vul phenotype caused by *mpk-1(lf)* (Table 3, rows 13–14). Taken together, our epistasis analysis indicates that DAF-18 inhibits RAS/MAPK signaling downstream of or in parallel with the RAS-GEF SOS-1 and upstream or at the level of the MAP kinase MPK-1.

**Table 3:** Epistasis analysis of *daf-18* with components of the RAS/MAPK pathway.

	genotype	Induction±SE	% Vul	% Muv	n
1	wild-type	3.00±0.00	0.0	0.0	many
2	*daf-18(lf)*	2.99±0.01	0.9	0.0	107
3	*lin-3(lf)*	0.81±0.19	95.2	0.0	21
4	*daf-18(lf) lin-3(lf)*	0.46±0.13	94.1	0.0	34
5	*let-23(rf)*	0.55±0.08	93.8	0.0	129
6	*let-23(rf); daf-18(lf)*	1.90±0.10*** ^(5)^	63.9	6.8	133
7	*lin-2(lf)*	1.34±0.19	73.7	0.0	42
8	*daf-18(lf); lin-2(lf)*	2.50±0.12*** ^(7)^	35.5	1.7	59
9	*let-60(gf); sos-1(lf)* #	2.60±0.17	16.7	0.0	30
10	*daf-18(lf) let-60(gf); sos-1(lf)* #	4.19±0.12*** ^(9)^	0.0	90.3	31
11	*lin-45(rf)*	1.81±0.15	57.3	0.0	68
12	*daf-18(lf) lin-45(rf)*	2.29±0.17* ^(11)^	39.5	2.3	42
13	*mpk-1(lf)*	0.00±0.00	100	0.0	14
14	*mpk-1(lf); daf-18(lf)*	0.00±0.00	100	0.0	17
15	*let-60(gf)* - gonad	2.44±0.27	46	23	13
16	*daf-18(lf) let-60(gf)* - gonad	3.38±0.38	8	42	12

SE indicates the standard error of the mean. % Vul indicates the fraction of animals with three or less induced VPCs. % Muv indicates the fraction of animals with more than three induced VPCs. Induction indicates the average number of induced VPCs per animal.

* indicates a p-value<0.05.

*** indicates a p-value<0.0005, numbers in brackets indicates the row number against which a t-test was performed. Alleles used: LG II: *let-23(sy1)*, LG III: *mpk-1(ga117)*, LG IV: *daf-18(ok480), let-60(n1046), lin-3(e1417), lin-45(sy96)*, LG V: *sos-1(s1031)*, LG X: *lin-2(n397)*.

# *sos-1(s1031)* is cis-linked to *unc-46(e177)*.

### DAF-18 negatively regulates MPK-1 but not MEK-2 phosphorylation

**Figure 2:**
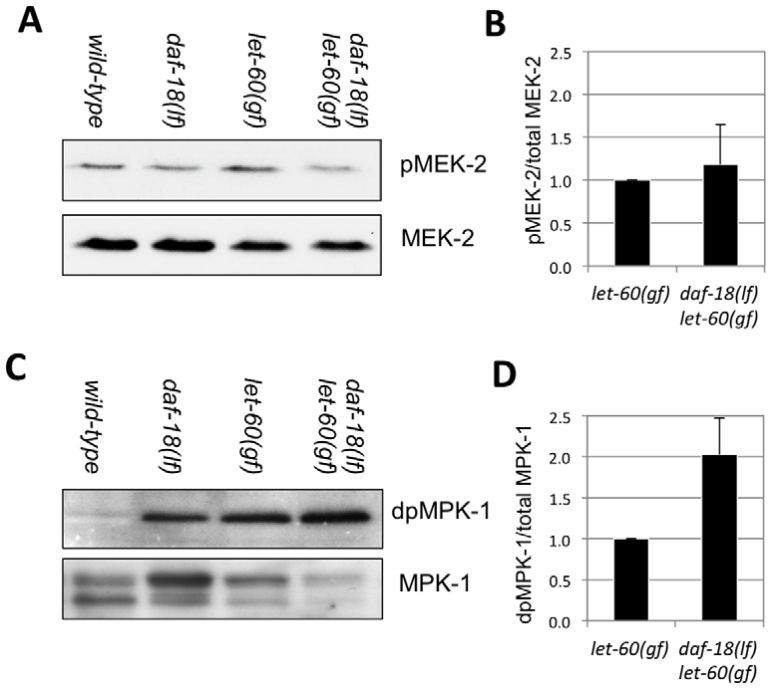
DAF-18 inhibits MPK-1 phosphorylation. Total extracts of wild-type, *daf-18(lf), let-60(gf)* and *daf-18(lf) let-60(gf)* larvae at the L4 stage were analyzed on Western blots using antibodies against (A) phosphorylated and total MEK-2 and (C) against di-phosphorylated (dp) and total MPK-1. Signal intensities were quantified in four (for MEK-2) and three (for MPK-1) independent experiments, and the average ratios of (B) pMEK-2 to total MEK-2 and (D) dpMPK-1 to total MPK-1 intensities were calculated.

Activation of the RAS/MAPK pathway results in an increased phosphorylation and activity of the downstream effectors RAF, MAPK kinase (MEK) and MAPK. We thus examined if *daf-18(lf)* mutants exhibit elevated levels of MEK and MAPK phosphorylation. Western blots of extracts from L4 larvae were probed with antibodies against mono-phosphorylated MEK (pMEK-2) and di-phosphorylated MAPK (dpMPK-1). Although the *C. elegans* genome encodes two MEK genes, MEK-1 and MEK-2, the phosphorylation site in human MEK to which the phospho-MEK antibody was raised (S217/S221) is only conserved in *C. elegans* MEK-2. Thus, we were able to specifically detect phosphorylated MEK-2 in whole animal extracts. Wild-type and *daf-18(lf)* L4 larvae contained only low levels of pMEK-2 and dpMPK-1 that could not be reliably quantified. As expected, *let-60(gf)* single mutants contained significantly higher levels of pMEK-2 and dpMPK-1 than wild-type larvae (Figure 2A and 2C). However, we observed no further increase in pMEK-2 levels in *daf-18(lf) let-60(gf)* double compared to *let-60(gf)* single mutants (Figure 2B). In contrast, dpMPK-1 levels were around two-fold increased *daf-18(lf) let-60(gf)* compared to *let-60(gf)* mutants (Figure 2D). Together with the genetic epistasis data presented above, the increase in MPK-1 phosphorylation in the absence of a significant change in MEK-2 phosphorylation indicates that DAF-18 most likely inhibits vulval induction at the level of MPK-1. Finally, the fact that we observed increased MPK-1 phosphorylation in total worm lysates suggests a global regulation of the RAS/MAPK pathway by DAF-18, probably including the germline.

### *daf-18* expression in the VPCs inhibits vulval induction

**Figure 3:**
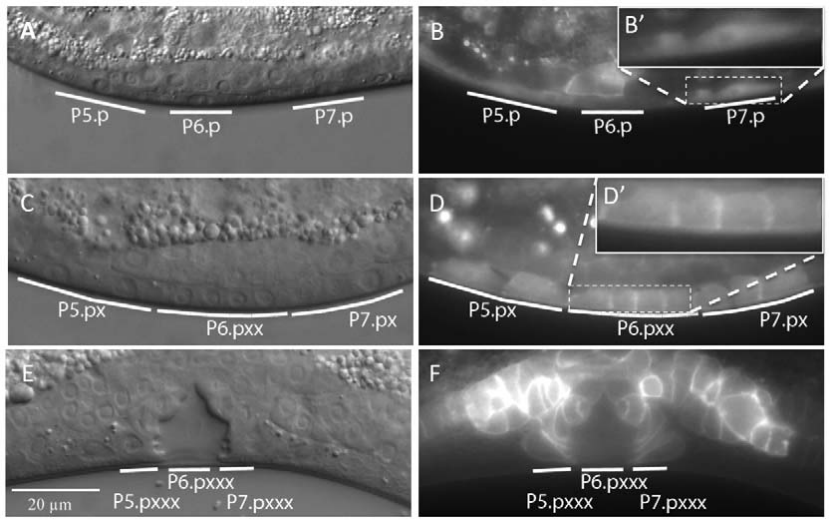
Expression pattern and sub-cellular localization of DAF-18::GFP. (A and B) Nomarski and fluorescence images of animals expressing the DAF-18::GFP translational reporter at the Pn.p cell stage, (C and D) the Pn.px(x) stage and (E and F) the Pn.pxxx “Christmas tree” stage. The insets in (B′) and (D′) show higher magnifications of the areas in (B) and (D) marked with dashed boxes.

**Figure 4:**
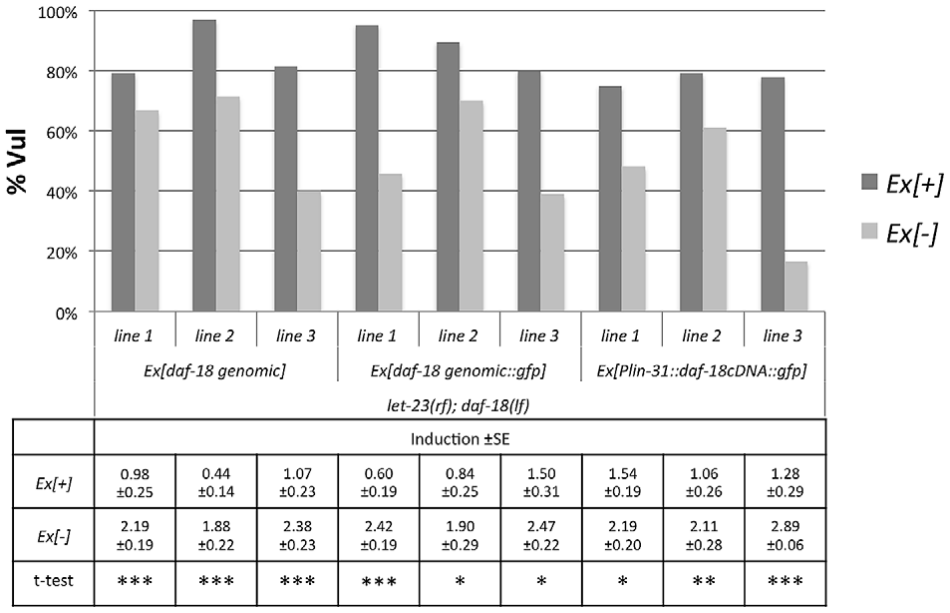
DAF-18 expression in the Pn.p cells inhibits vulval induction. Vulval induction was scored at the L4 stage comparing *let-23(rf); daf-18(lf)* animals carrying the different extra-chromosomal arrays (Ex[+]) to their siblings lacking the array (Ex[−]). Three independent lines were scored per construct and at least 20 Ex[+] and Ex[−] animals were counted per line. For each line, a t-test was performed comparing induction in Ex[+] and Ex[−] animals on the same plates. * indicates a p-value≤0.05, ** a p-value≤0.005, and *** a p-value≤0.0005.

To further investigate the role of DAF-18 during vulval induction, we constructed a translational reporter by inserting a *gfp* cassette at the 3′ end of the ORF in a genomic *daf-18* fragment (Figure 3). This DAF-18::GFP reporter rescued both the dauer defective (DAF-d) phenotype (data not shown) as well as the vulval phenotypes of *daf-18(lf)* with similar efficiency as a 6.5 kb genomic fragment spanning the entire *daf-18* locus (Figure 4). DAF-18::GFP expression was observed in many tissues during all larval stages, including the developing vulva, the uterus, the ventral nerve cord and the distal tip cells (data not shown). In particular, equal levels of DAF-18::GFP expression were detected in the six VPCs of L2 larvae, and expression persisted in the descendants of the induced VPCs until the Pn.pxxx stage (Figure 3). Interestingly, the sub-cellular localization of DAF-18::GFP changed over the course of vulval development. In the VPCs of L2 larvae prior to and during induction (Pn.p stage), DAF-18::GFP was predominantly localized in the cytoplasm and the nucleus (Figure 3A, 3B and 3B′). However, at the subsequent stages (Pn.px to Pn.pxx stages), DAF-18::GFP became increasingly localized to the plasma membrane of the vulval cells (Figure 2C, 2D and 2D′). Plasma membrane staining peaked at the “Christmas tree” (Pn.pxxx) stage, when almost all the protein appeared to be localized to the membranes and nuclear staining was reduced to very low levels (Figure 3E and 3F). Since the DAF-18::GFP reporter was also expressed in tissues surrounding the vulval cells, we examined whether tissue-specific expression of DAF-18 in the VPCs reduces vulval induction. For this purpose, we expressed *daf-18 cDNA* fused to *gfp* under the control of the Pn.p cell-specific *lin-31* promoter, which is active in the VPCs before and during vulval induction [29] (*Plin-31::daf-18 cDNA::gfp::unc-54 3′ UTR*). Indeed, introduction of the *lin-31::daf-18::gfp* transgene into *daf-18(lf); let-23(rf)* animals repressed vulval induction with similar efficiency as the *daf-18::gfp* reporter or a genomic *daf-18* rescue construct (Figure 4).

Besides the vulval cells, the DAF-18::GFP reporter was also expressed at the L3 to L4 larval stages in several cells of the uterus, which is part of the somatic gonad (Figure 3). We thus tested if the *daf-18*-mediated repression of vulval induction requires the gonad by ablating the Z1 to Z4 somatic gonad and germline precursor cells at the L1 stage. In gonad-ablated *daf-18(lf) let-60(gf)* double mutants, vulval induction was higher than in gonad-ablated *let-60(gf)* single mutants, indicating that DAF-18 represses vulval induction independently of a signal from the gonad (Table 3, rows 15, 16). Thus, DAF-18 probably acts predominantly in the VPCs to inhibit MAPK signaling during vulval induction.

### Both lipid and protein phosphatase activities of DAF-18 inhibit vulval induction

**Figure 5:**
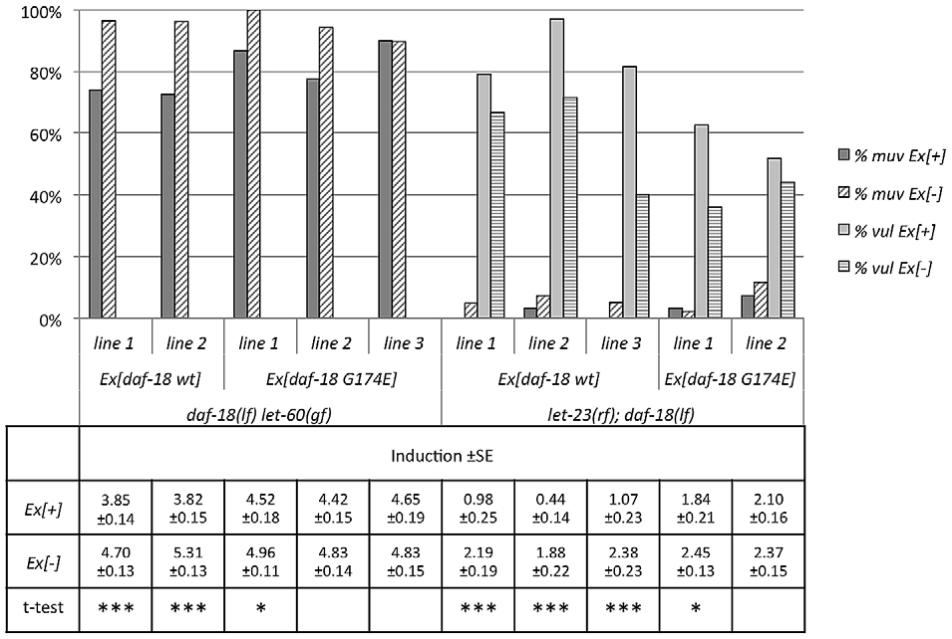
A lipid phosphatase deficient mutant of DAF-18 remains partially active. Animals carrying the *daf-18 wt and daf-18 G174E* extra-chromosomal arrays were scored at the L4 stage as described in the legend to Figure 4.

Mammalian PTEN acts as a lipid phosphatase as well as a dual-specificity protein phosphatase [5], [6]. Moreover, a recent report has shown that *C. elegans* DAF-18 can act as a protein phosphatase inhibiting signaling by the VAB-1 ephrin receptor during oocyte maturation [18]. The G129E mutation in the catalytic center of human PTEN eliminates the lipid phosphatase activity, while retaining the protein phosphatase activity [7]. The corresponding glycine 174 residue in *C. elegans* DAF-18 was therefore mutated to glutamic acid in the *daf-18 genomic* rescue construct. To quantify the rescuing activity of the *daf-18* wild-type (*daf-18 wt*) and the G174E mutated lipid phosphatase mutant (*daf-18 G174E*), these constructs were expressed in the *daf-18(lf) let-60(gf)* and *let-23(rf); daf-18(lf)* backgrounds, and vulval induction was quantified. As expected, expression of *daf-18 wt* rescued both the DAF-d (data not shown) and vulval phenotypes of *daf-18(lf)* (Figure 5). In contrast, *daf-18 G174E* did not rescue the DAF-d phenotype ([15] and own observation), yet exhibited a weaker, though significant rescuing activity of the vulval induction phenotype (Figure 5). These results indicate that the DAF-18 protein and lipid phosphatase activities each play independent roles in negatively regulating the RAS/MAPK pathway and that both activities are required for the full inhibition of vulval induction by DAF-18.

## Discussion

**Figure 6:**
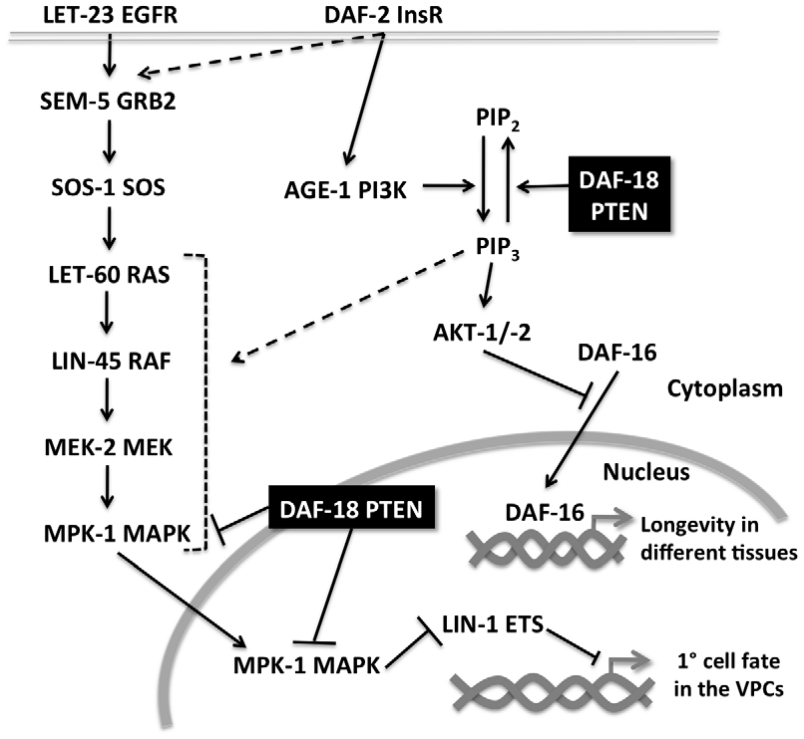
Multiple modes of crosstalk between the RAS/MAPK and insulin pathways during vulval development. Activation of LET-60 RAS in the VPCs causes phosphorylated MPK-1 to enter the nucleus where it phosphorylates transcription factors such as LIN-1 ETS, which represses 1° fate-specific gene expression. The insulin signaling pathway interacts with the RAS/MAPK pathway at several levels. Firstly, DAF-2 InsR activation enhances RAS/MAPK signaling by stimulating the PI3K AGE-1, which positively regulates RAS/MAPK signaling via increased PIP_3_ production. In addition, DAF-2 signaling activates the RAS/MAPK signaling independently of AGE-1, possibly by recruiting SEM-5 to the plasma membrane. DAF-18 PTEN inhibits RAS/MAPK signaling in two distinct manners; by dephosphorylating PIP_3_ and by negatively regulating MAPK activation.

The Insulin pathway is a key regulator of development, reproduction, and life span in metazoans. In this study, we have discovered a new form of cross-talk between the Insulin and RAS/MAPK pathways during vulval development. Signaling by the Insulin receptor DAF-2 positively regulates MAPK activation. Surprisingly, the effect of DAF-2 on vulval development does not involve activation of the canonical Insulin pathway. DAF-2 signaling regulates vulval induction in at least two distinct manners, through AGE-1 dependent and independent pathways (Figure 6). One possible explanation for the AGE-1-independent branch of DAF-2 signaling is supported by mammalian data, which suggest that the Insulin receptor can directly stimulate RAS activation by recruiting GRB2 and the RAS-GEF SOS [30], [31]. Also in *C. elegans*, LET-60 RAS was found to act downstream of the DAF-2 Insulin receptor to modulate the effects of Insulin signaling during entry into the Dauer stage [32].

Furthermore, we found that the PTEN ortholog DAF-18 strongly inhibits RAS/MAPK signaling. Vulval induction in *daf-18(lf) let-60(gf)* double mutants reaches levels similar to those seen in the strongest Muv mutants such as *lin-15AB(lf)* [33]. The increase in RAS/MAPK signaling in *daf-18(lf)* mutants could be partially reverted by loss of the PI3K activity, suggesting that elevated levels of PIP_3_ do stimulate RAS/MAPK signaling but cannot explain all the functions DAF-18 exerts during vulval induction. Accordingly, the inhibitory activity requires both the lipid and protein phosphatase activities of DAF-18. PIP_3_ acts as a second messenger that activates multiple downstream targets. One major PIP_3_ target in the Insulin pathway is the AKT kinases, which phosphorylate and thereby inhibits the FOXO transcription factor DAF-16. However neither *akt-1, akt-2* nor *daf-16* mutations had any detectable effect on vulval induction. Thus, PIP_3_ must act via other targets to stimulate RAS/MAPK signaling. Increased levels of PIP_3_ in the plasma membrane could, for example, enhance the recruitment of an alternative GEF that activates RAS signaling in parallel to the RAS-GEF SOS-1 [28] (Figure 6). However, we observed that prior to and at early stages of vulval induction, DAF-18::GFP was localized predominantly in the cytoplasm and nucleus of the VPCs, while membrane localization of DAF-18 only became apparent at later stages. Previous observations of mammalian PTEN localization suggested that PTEN performs different functions depending on its sub-cellular localization [34]. It has been proposed that the lipid phosphatase activity is important for the cytoplasmic and membrane functions of mammalian PTEN, while the protein phosphatase activity is rather required for its nuclear functions [34], [35]. The nuclear localization of PTEN in mammalian cells is often associated with cell-cycle arrest in G1 and accompanied by decreased levels of ERK phosphorylation. Prior to vulval induction, the VPCs are maintained in a long G1 arrest lasting the entire L2 stage [36]. It is therefore possible that initially DAF-18 acts predominantly in the nucleus as a protein phosphatase that negatively regulates vulval induction. Indeed, Western blot analysis revealed elevated levels of dpMPK-1 in *daf-18(lf)* mutants, supporting our model that DAF-18 –directly or indirectly- blocks MAPK activation (Figure 6).

In humans, PTEN is one of the most frequently mutated tumor suppressor genes. However, not all disease phenotypes associated with loss of PTEN can be explained by hyper-activation of the Insulin pathway alone. Thus, PTEN must have other functions that are independent of its inhibitory activity in the Insulin pathway. Accordingly, Suzuki and Han [19] observed many synthetic phenotypes in *C. elegans daf-18(lf)* mutants, including embryonic lethality and sterility, which are independent of DAF-16 FOXO and do not involve DAF-2 InsR signaling. Our work highlights the importance of *C. elegans* DAF-18 PTEN in regulating a range of biological processes and may serve as a basis to better understand the multiple roles human PTEN plays during cancer initiation and progression. Thus, single mutations in the PTEN tumor suppressor may result in the simultaneous hyper-activation of several oncogenic signaling pathways.

## Materials and Methods

### General worm methods

Standard methods were used for maintaining and manipulating *Caenorhabditis elegans* [37]. Animals were cultured at 20°C and the wild-type strain is the Bristol N2 strain. Information regarding the mutants used in this study can be found on WormBase (http://www.wormbase.org). Mutations used according to their linkage group:

LG I: *daf-16(mu86)*, LG II: *age-1(mg44), let-23(sy1)*, LG III: *daf-2(e1370), mpk-1(ga117)*, LG IV: *let-60(n1046), daf-18(ok480), lin-3(e1417), lin-45(sy96)*, LG V: *akt-1(mg144gf), akt-1(ok525lf)*, LG X: *lin-2(n397), sos-1(s1031), unc-46(e177)* to cis link *sos-1(s1031)*, LG X: *lin-2(n397), gap-1(ga133)*. Transgenes used: *syIs59[Pegl-17::cfp], zhIs4[Plip-1::gfp], zhEx382[daf-18 genomic] zhEx343[daf-18::gfp], zhEx358[Plin-31::daf-18::gfp], zhEx344[daf-18 G174E]*.

### Plasmids and PCR fusion constructs

pIN05 (*daf-18 genomic wt*) was made by cloning the whole genomic fragment of *daf-18* starting 1.3 kb upstream of the ATG and ending 0.5 kb downstream of the STOP and cloning to pGEM-T. pIN03 (*daf-18 genomic G174E*) was made by fusion PCR [38] of two overlapping fragments of the whole genomic *daf-18* starting 1.3 kb upstream of the ATG and ending 0.5 kb downstream of the STOP using primers which contain the mutation G174E (GGC to GAA) in the overlapping region and cloning to pGEM-T. pIN17 (*Plin-31::daf-18 cDNA::gfp::unc-54 3′UTR*) was made by amplifying *daf-18 cDNA::gfp* from a previously cloned plasmid with primers containing NotI sites on both ends, digestion with NotI and cloning into the NotI site of the pB253 plasmid containing the *lin-31* enhancer and promoter. The *daf-18 genomic* translational GFP reporter was made using fusion PCR of three parts by inserting a *gfp* cassette in frame between the last exon and the 3′ UTR into a genomic fragment encompassing 1.3 kb of 5′ regulatory sequences and the complete *daf-18* coding sequences. Sequences of the primers used for the different constructs can be found in Table S1.

### Transgenic lines

Worms expressing extra-chromosomal transgenic arrays were generated by microinjection of DNA into young adult worms [39]. pIN03 (*zhEx344*), pIN05 (*zhEx382*) and pIN17(*zhEx358*) were injected at a concentration of 50 ng/µl. The fusion PCR *daf-18 genomic::gfp* (*zhEx343*) translational reporter was injected at a concentration of 30 ng/ul. Co-markers used were either pCFJ90 (Pmyo-2::mCherry) at a concentration of 2 ng/ul or pTJ1157 (Plin-48::gfp) at a concentration of 50 ng/ul. Final concentration of injected DNA was filled up to 150 ng/ul using the empty plasmid pBluescript-KS.

### Fluorescence microscopy

DAF-18::GFP, P*lip-1*::GFP and P*egl-17*::CFP expression were observed under fluorescent light illumination with either a Leica DMRA microscope equipped with a cooled CCD camera (Hamamatsu ORCA-ER) or Olympus BX61 with Q Imaging Fast 1394 Retiga 2000R camera (Q Imaging Inc., Canada) controlled by the Openlab 5.x software (Improvision/PerkinElmer). Animals were mounted on 4% agarose pads in M9 solution with 20 mM tetramisole hydrochloride. Quantification of fluorescence levels was performed under the same picture acquisition settings for all conditions examined.

### Vulval induction

Vulval induction was scored by examining worms at the L4 stage under Nomarski optics as described [40]. The number of VPCs that had adopted a 1° or 2° Vulval fate was counted for each animal and the induction index was calculated by dividing the number of 1° or 2° induced cells by the number of animals scored. Statistical analysis was performed using a t-test for independent samples.

### RNA interference analysis

RNA interference analysis (RNAi) was performed by feeding animals dsRNA-producing bacteria as described previously [41]. Around 10 to 20 P0 animals at the L1 larval stage were transferred to plates containing RNAi bacteria grown on 3 mM IPTG. Vulval induction was scored in the F1 generation (or the P0 for *akt-2* RNAi) at the L4 larval stage to count the number of induced VPCs. *gfp, akt-2, piki-1* and *vps-34* RNAi clones were all taken from the Ahringer RNAi library.

### Western blot analysis

Forty-five animals at the L4 stage were placed into 15 µl of 1× SDS sample buffer, lysed at 95°C for 5 min, centrifuged at 14,000 rpm for 2 min and the supernatant was loaded on 10% acrylamide gels, which were analyzed by Western blotting. Anti-phospho-MEK1/2 (S217/S221) and Anti-MEK1/2 (D1A5) antibodies were purchased from Cell Signaling Technology (Beverly, MA). Anti-di-phosphorylated ERK-1&2 (M8159) antibody was purchased from Sigma-Aldrich (St. Louis, MO), and anti-ERK 2 (K-23) antibody was purchased from Santa Cruz Biotechnology (Santa Cruz, CA). Quantification of the bands was performed using the gel quantification plugin in ImageJ software [42]. The ratios between phosphorylated and total MEK-2 and MPK-1 levels, respectively, were calculated and normalized for each independent experiment to the ratios measured in the *let-60(gf)* single mutants.

## Supporting Information

Table S1 Primers used to generate the plasmids described in the manuscript.
(DOC)
