# Supplementary material for: PTEN Negatively Regulates MAPK Signaling during Caenorhabditis elegans Vulval Development
Source: PLoS Genet. 2012 Aug 16;8(8):e1002881. doi: 10.1371/journal.pgen.1002881 (PMC3420937; doi:10.1371/journal.pgen.1002881)
Supplement: Table S1 — Primers used to generate the plasmids described in the manuscript. (DOC) [file pgen.1002881.s001.doc]

| Primer | sequence | Comments | construct |
| --- | --- | --- | --- |
| OIN22 | cgtcgatttttccgacttgt | *daf-18* 5’UTR forward | pIN05  pIN03 |
| OIN21 | aaaacaatgtaaattcgaatgcgcc | *daf-18* 3’UTR reverse | pIN05 pIN03 |
| OIN90 | catcactccggtacgttcttttccagc | *daf-18* G174E mutation forward. Used with OIN21 | pIN03 |
| OIN91 | gaaaagaacgtaccggagtgatgatatgtgctc ttctcatctacatc | *daf-18* G174E mutation fusion primer reverse. Used with OIN22 | pIN03 |
| OIN135 | tttgcggccgcatggttactcctcctccagatgt gcc | *daf-18* 5’ORF forward with NotI site | pIN17 |
| OIN136 | tttgcggccgcgctatttgtatagttcatcc | *gfp* 3’ORF reverse with NotI site | pIN17 |
| OIN50 | cggattcgaattttgatcaagctatttatttgag cttgcatgcctgcaggtcgact | *gfp* 5’ORF forward with overlap to *daf-18* 3’ORF. Fusion primer | *daf-18* *genomic::gfp* fusion |
| OIN98 | ggatgaactatacaaatagcgattttcttctta ctgaccctcc | *daf-18* 3’UTR forward with overlap to *gfp* 3’ORF. Fusion primer | *daf-18* *genomic::gfp* fusion |

Suppl. Table s1. Primers used to generate the plasmids described in the manuscript.
